# Supplementary material for: Calreticulin and integrin alpha dissociation induces anti-inflammatory programming in animal models of inflammatory bowel disease
Source: Nat Commun. 2018 May 17;9:1982. doi: 10.1038/s41467-018-04420-4 (PMC5958137; doi:10.1038/s41467-018-04420-4)

SUPPLEMENTARY INFORMATION

Dissociation of calreticulin and integrin alpha induces anti-inflammatory programming in animal models of inflammatory bowel disease.

Masayoshi Ohkuro†, Jun-Dal Kim†, Yoshikazu Kuboi, Yuki Hayashi, Hayase Mizukami, Hiroko Kobayashi-Kuramochi, Kenzo Muramoto, Manabu Shirato, Fumiko Michikawa-Tanaka, Jun Moriya, Teruya Kozaki, Kazuma Takase, Kenichi Chiba, Kishan Lal Agarwala, Takayuki Kimura, Makoto Kotake, Tetsuya Kawahara, Naoki Yoneda, Shinsuke Hirota, Hiroshi Azuma, Nobuko Ozasa-Komura, Yoshiaki Ohashi, Masafumi Muratani, Keiji Kimura, Ieharu Hishinuma and Akiyoshi Fukamizu*

† These two authors contributed equally to this work.

* Correspondence to: Dr. Akiyoshi Fukamizu
Life Science Center for Survival Dynamics, Tsukuba Advanced Research Alliance (TARA), University of Tsukuba, 1-1-1 Tennodai Tsukuba, Ibaraki 305-8577, Japan.
TEL: +81-29-853-6070, FAX: +81-29-853-6070
E-mail: akif@tara.tsukuba.ac.jp

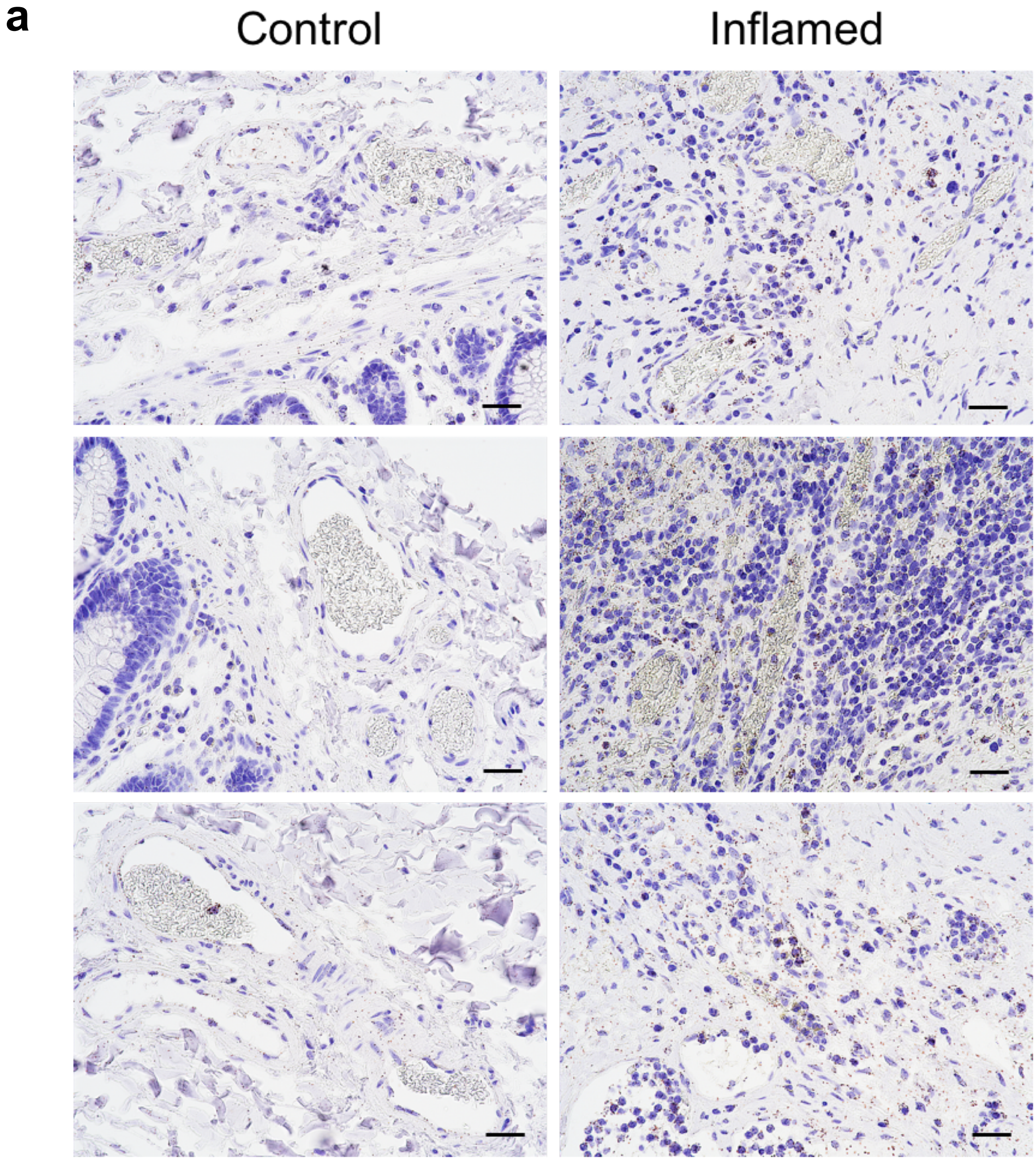

Supplementary Figure 1a. ***In situ*** **PLA assay for the interaction between CRT and ITGA4 at the vascular lumen and mucosa in the UC colon (Patient #1).** Representative images; non-inflamed control (left) and the inflamed site (right). Scale bars, 20 μm.

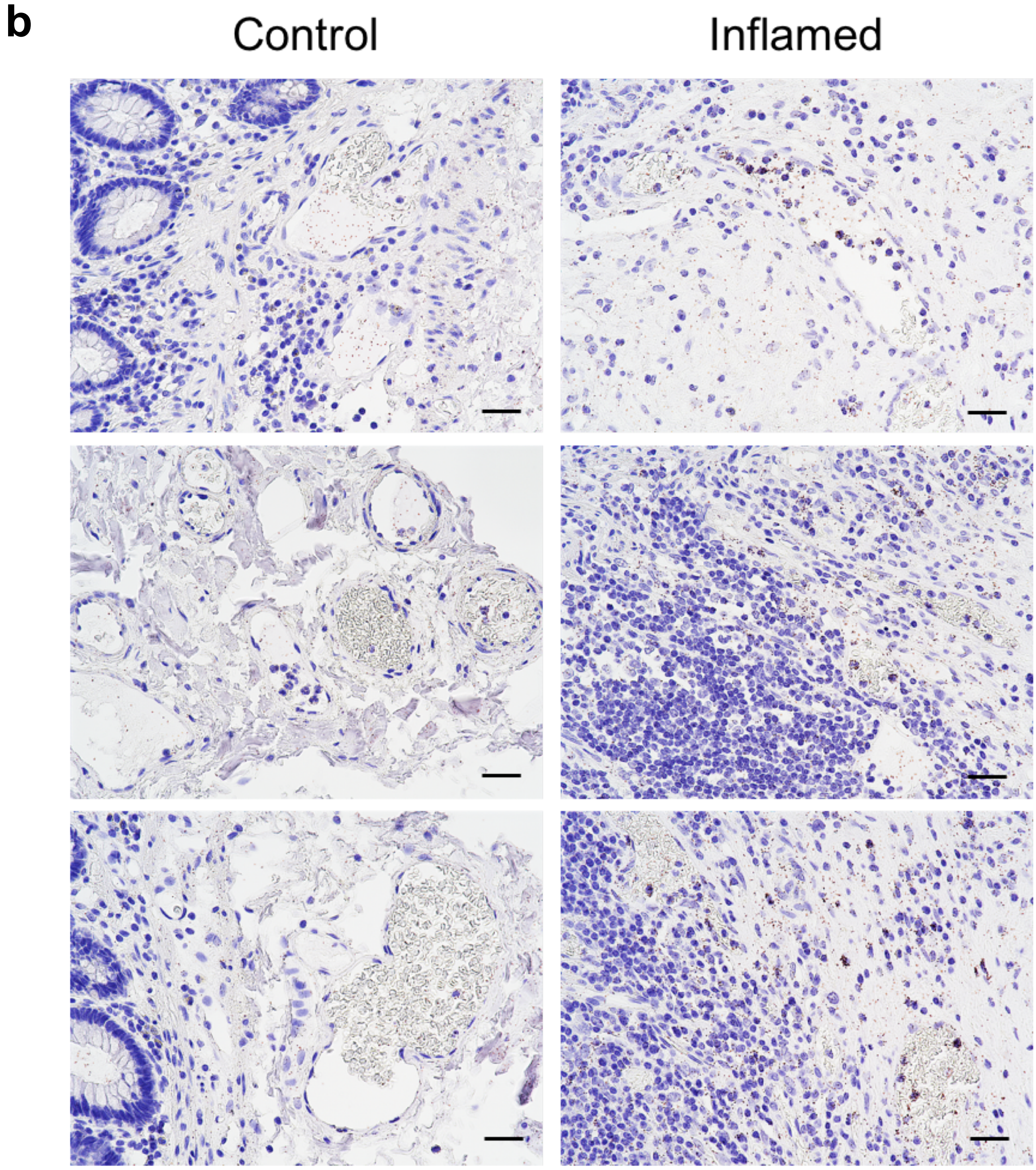

Supplementary Figure 1b. *In situ* PLA assay for the interaction between CRT and ITGA4 at the vascular lumen and mucosa in the UC colon (Patient #2). Representative images; non-inflamed control (left) and the inflamed site (right). Scale bars, 20 μm.

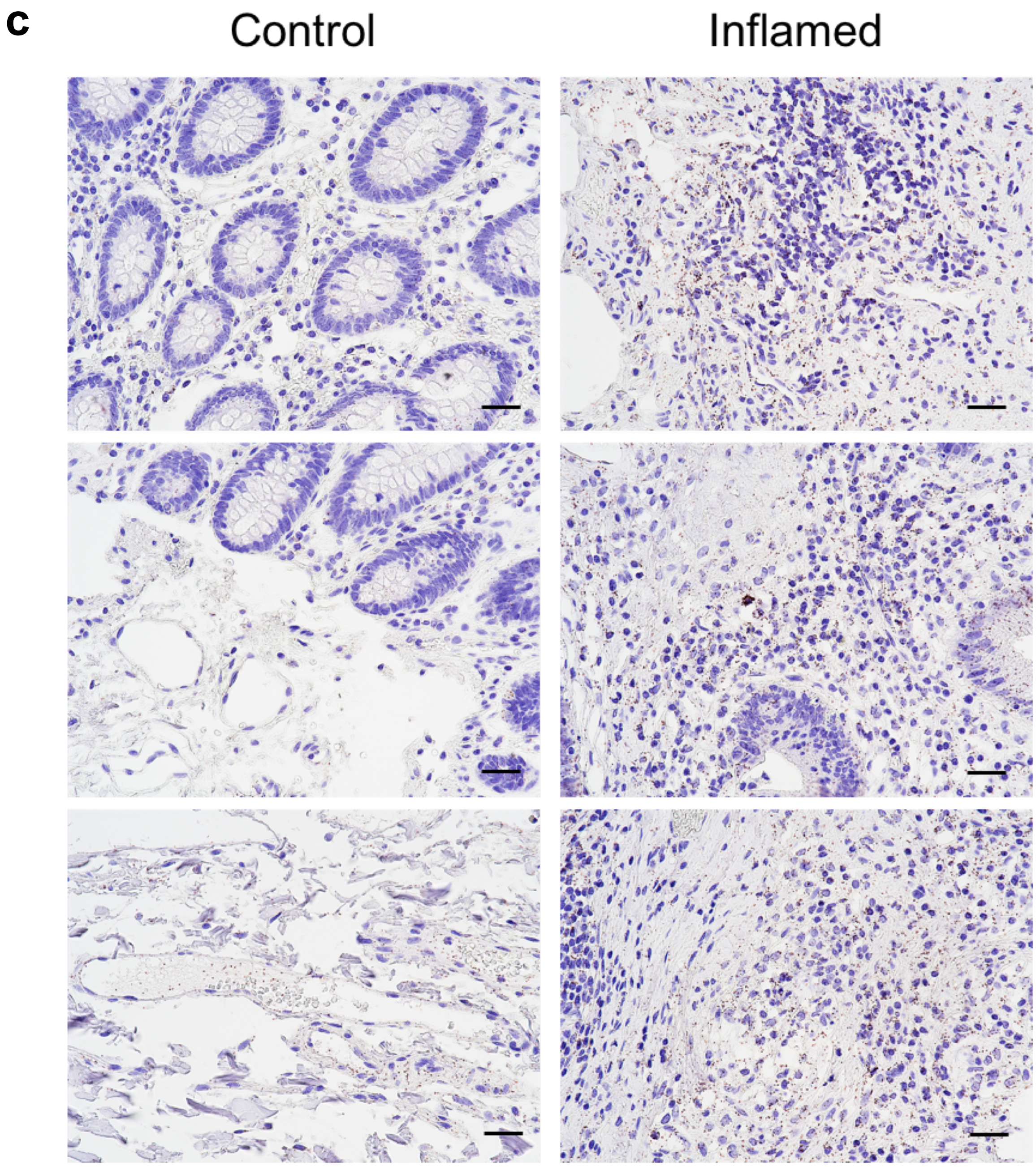

Supplementary Figure 1c. ***In situ*** **PLA assay for the interaction between CRT and ITGA4 at the vascular lumen and mucosa in the UC colon (Patient #3).** Representative images; non-inflamed control (left) and the inflamed site (right). Scale bars, 20 μm.

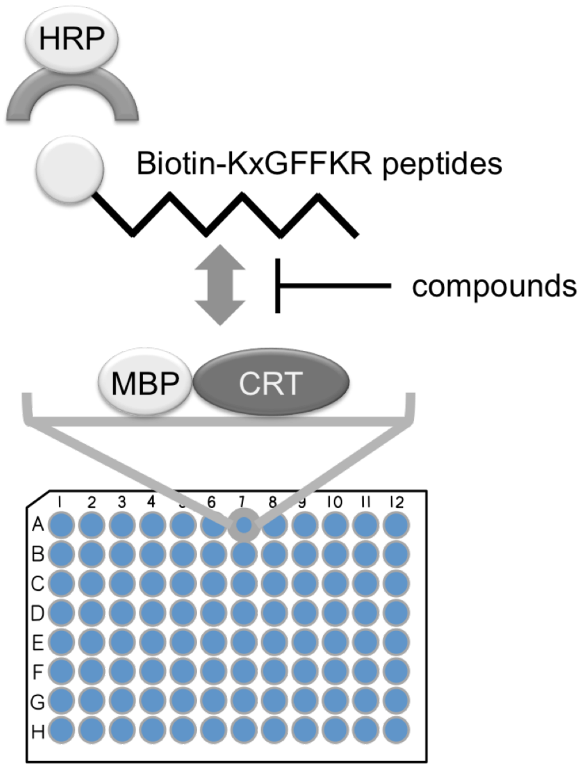

Supplementary Figure 2. Cell-free binding assay-based high-throughput screening system. Schematic illustration of the HTS cell-free system. Each biotinylated α integrin peptide was added to a CRT-coated 96-well plate, and peptides bound to CRT were detected with streptavidin–horseradish peroxidase (HRP).

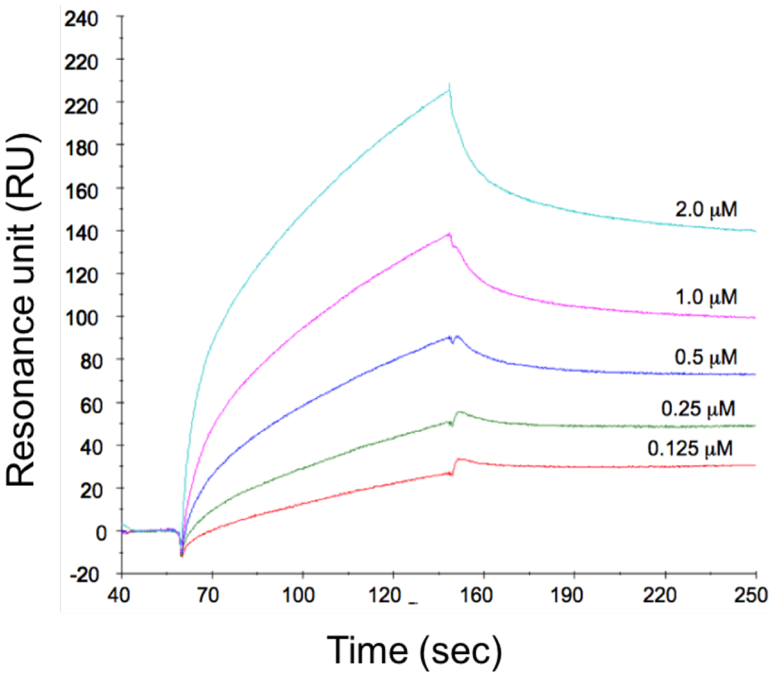

Supplementary Figure 3. Overlay plot of sensorgrams of the CRT–ITGA4 interaction. The biotinylated intracellular domain of ITGA4 peptide was immobilized on a streptavidin-coated chip. CRT at 0.125, 0.25, 0.5, 1.0 and 2.0 μM was injected onto the sensor chip, and CRT binding to the immobilized ITGA4 was analyzed. Retention of CRT on the sensor chip was indicated by a change in resonance units (RU) over the course of the 120 s injection interval. Binding affinity, referred to as the dissociation constant K_D value was determined by fitting the sensorgram with the global Langmuir 1:1 binding model allowed determination of the association (k_a) and dissociation (k_d) rate constants for CRT binding to the ITGA4 peptide. k_a and k_d rate constants were obtained as 1.54 x 10^4 $M^{-1}s^{-1}$ and 3.88 x 10^{-3} s^{-1}, respectively, and thus the dissociation constant of K_D (k_d / k_a) was calculated as 2.5 x 10^{-7}.

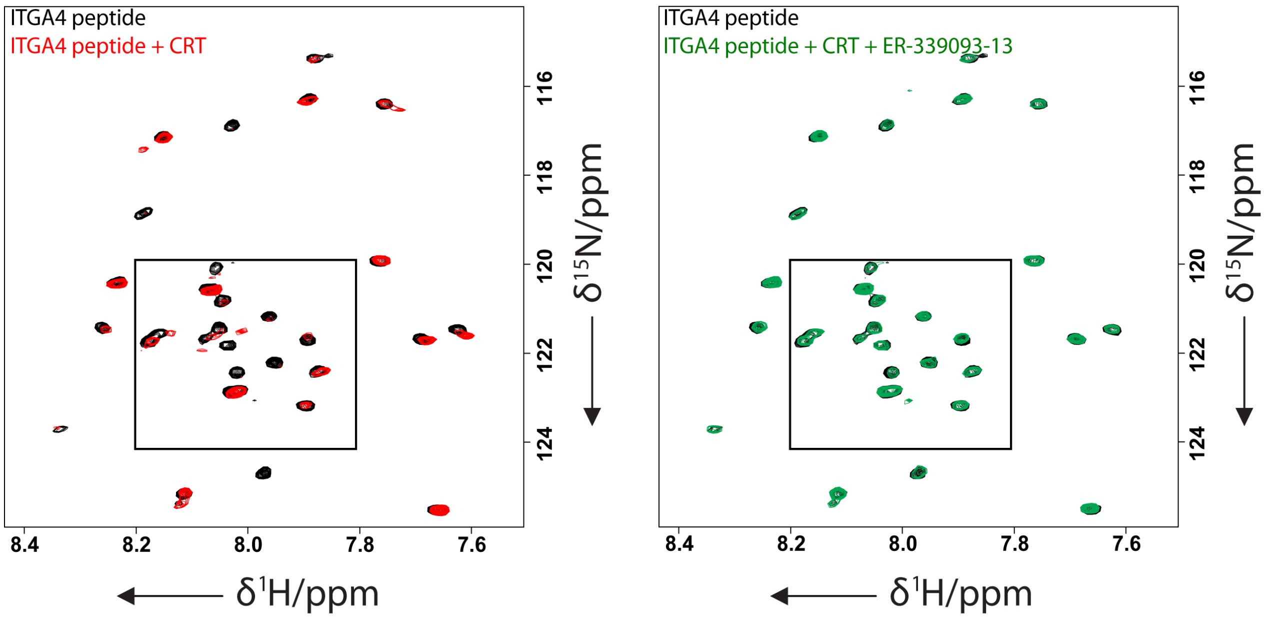

Supplementary Figure 4. HSQC spectra of ITGA4 peptides with CRT or CRT+ER-339093-13. Overall view of the ^{15}N-HSQC spectra in Fig. 1e. Two dimensional ^{1}H-^{15}N HSQC spectrum of the intracellular domain of ITGA4 peptides (black), with the CRT (red), and with combination of CRT and ER-339093-13 (green).

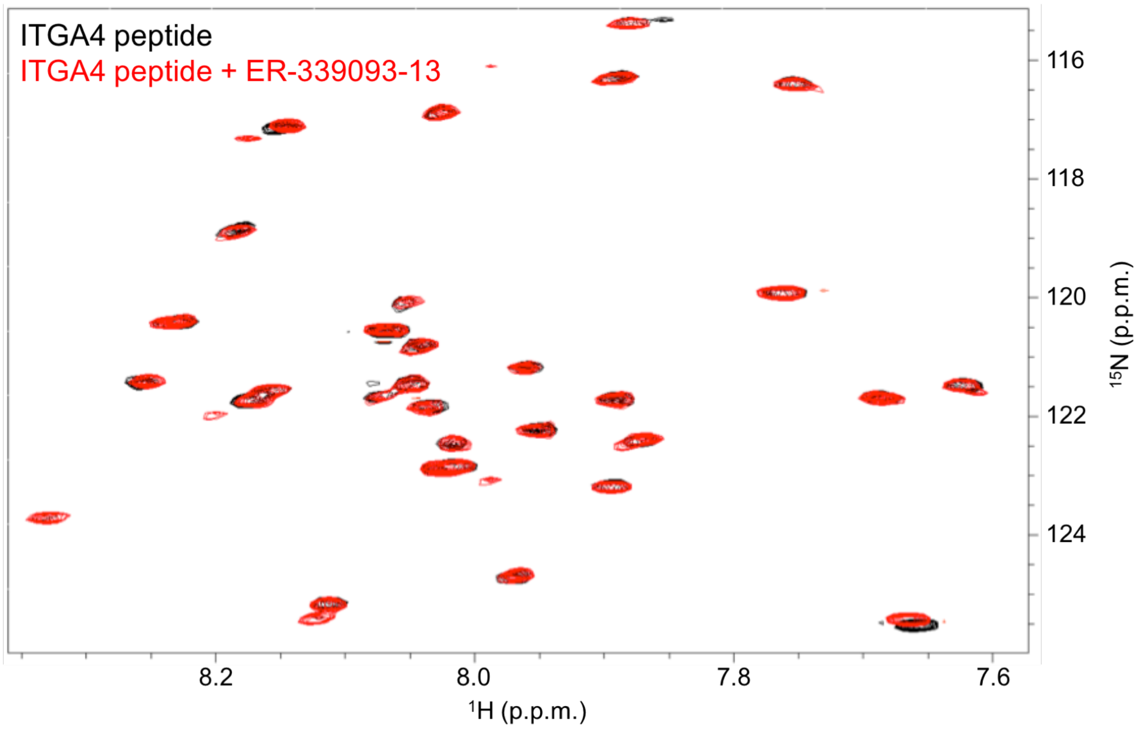

Supplementary Figure 5. HSQC spectra of ITGA4 peptides with ER-339093-13. Interaction between ER-339093-13 and α integrin was analyzed by comparing the HSQC spectrum of the ^{15}N-labeled intracellular domain of peptide (KAGFFKRQYKSILQEENRRDSWSYINSKSNDD) in the absence (black) or presence (red) of ER-339093-13 using a molar ratio of ITGA4 peptide to ER-339093-13 of 1:25. Two-dimensional ^{1}H-^{15}N HSQC spectra of ITGA4 peptide were collected with 128 scans and the complex points 128 (^{15}N) $\times$ 1024 (^{1}H).

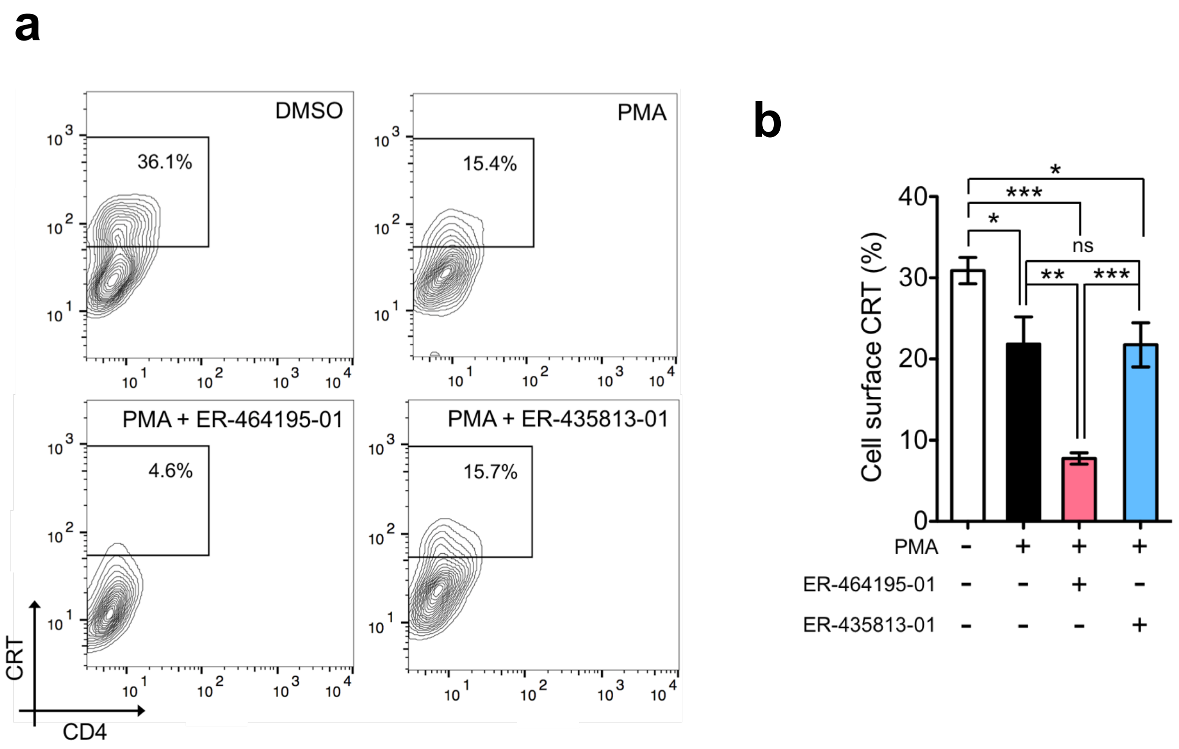

Supplementary Figure 6. Effect of ER-464195-01 on cell surface expression of CRT in Jurkat cells. **a**, Flow cytometric analysis for the cell surface CRT in PMA-stimulated Jurkat cells with ER-464195-01 or ER-435813-01. **b**, Quantitative analysis of (**a**), Results are given as mean ± SEM of n = 6 experiments; *$P < 0.05$, **$P < 0.01$, ***$P < 0.001$ and ns (not significant). Statistical significance was evaluated using one-way ANOVA with Bonferroni's multiple comparison test.

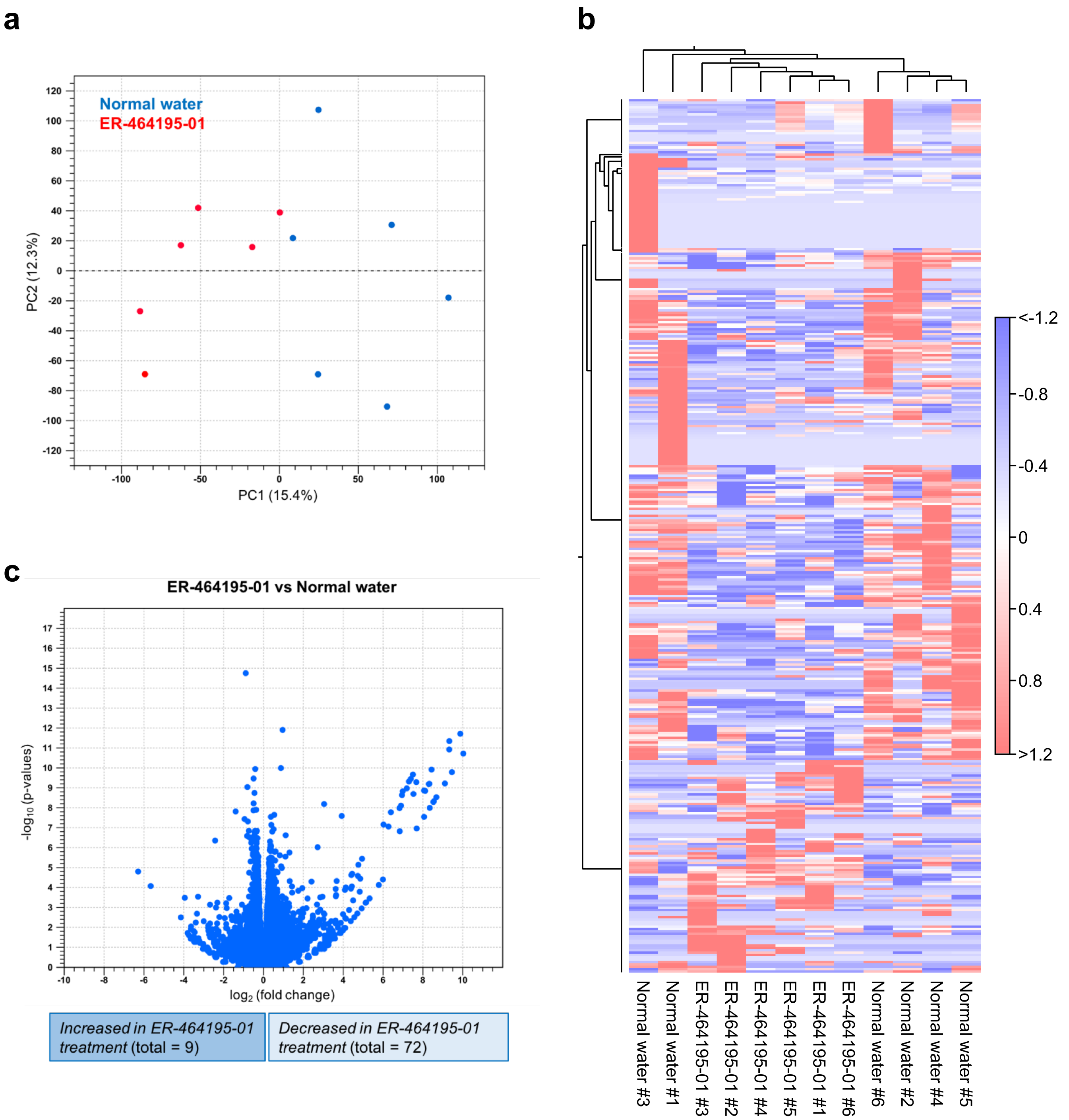

Supplementary Figure 7. Gene expression profile of RNA-Seq data distinguishing two groups. (**a**) Principal component analysis (PCA) was performed for RNA-seq data for control (normal water) and ER-4641945-01 matching samples, PC1 (x-axis, 15.4%) and PC2 (y-axis, 12.3%). (**b**) Hierarchical clustering of the expression profiles of differentially expressed between control and ER-4641945-01 treatment group (adjusted FDR *P* value < 0.05). (**c**) A volcano plot of differentially expressed. Full gene lists are in Supplementary Table 3.

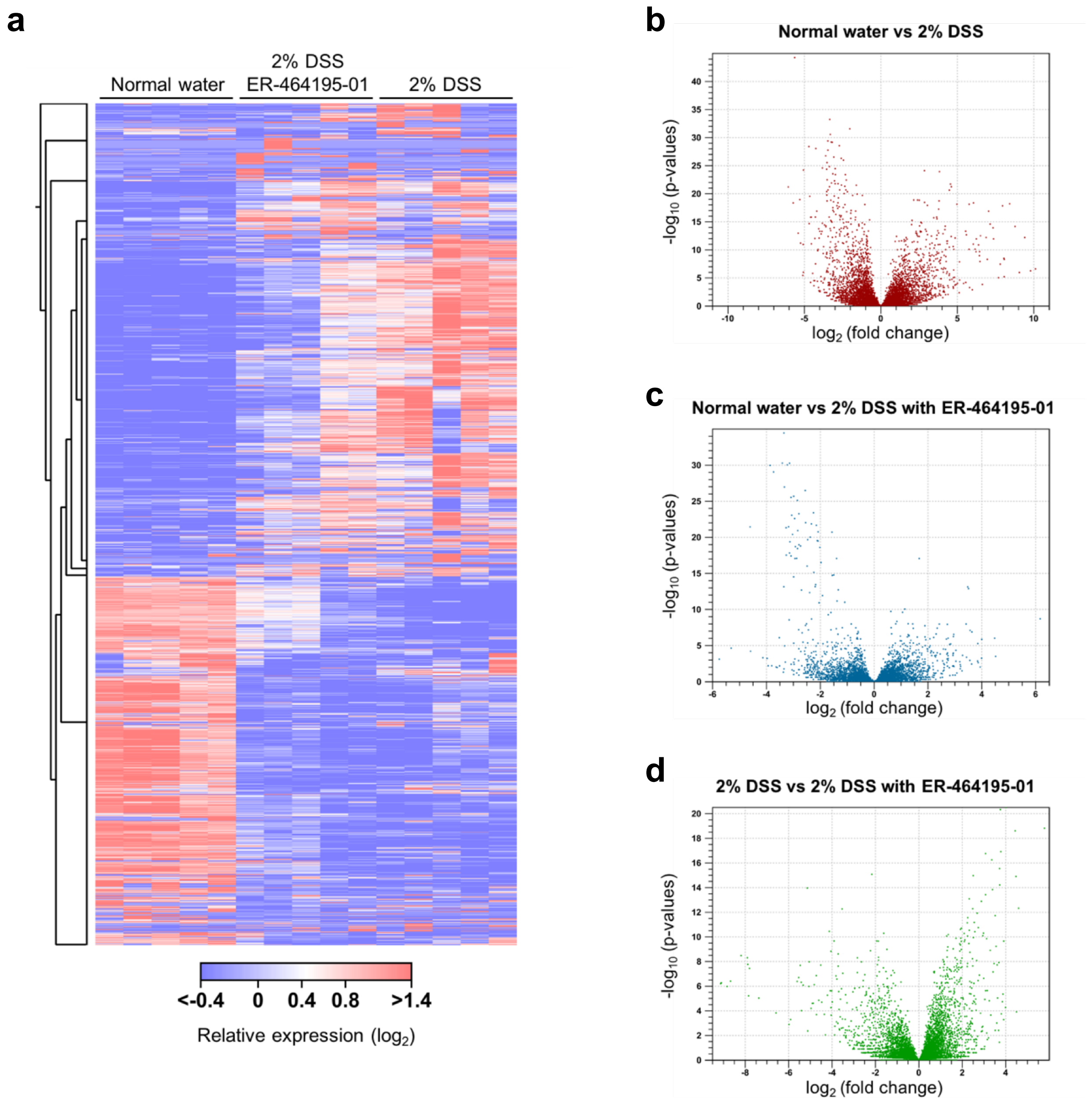

Supplementary Figure 8. Gene expression profile of RNA-Seq data distinguishing three groups. (**a**) Hierarchical clustering of the expression profiles of differentially expressed among control (normal water), 2% DSS group and 2% DSS with ER-4641945-01 group (adjusted FDR *P* value < 0.05). (**b**) A volcano plot of differentially expressed transcripts between 2% DSS and control group, and (**c**) between 2% DSS with ER-4641945-01 and control group, and (**d**) between 2% DSS with ER-4641945-01 and 2% DSS group, respectively.

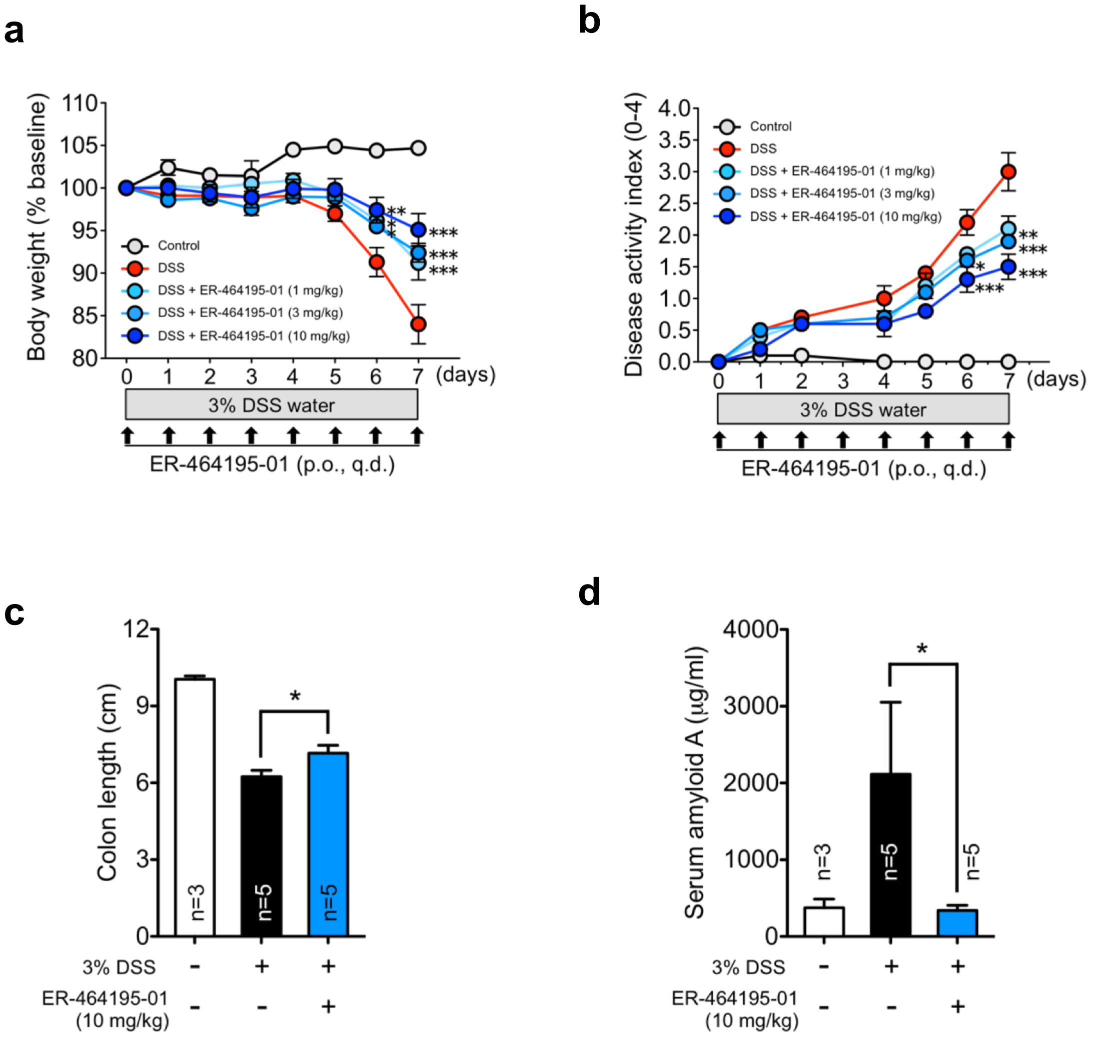

Supplementary Figure 9. Dose-dependent preventive effect of ER-464195-01 in colitis of 3% DSS-treated mice. Prophylactic treatments of DSS-treated mice with ER-464195-01 at doses of 1, 3 and 10 mg/kg. Body weight (**a**), disease activity index (DAI) (**b**), colon length (**c**) and concentration of SAA in sera (**d**). Results are given as the mean ± SEM of n = 3 or 5; $^{*}P < 0.05$, $^{**}P < 0.01$ and $^{***}P < 0.0001$ versus control group (two-way ANOVA followed with Bonferroni's post test) (**a** and **b**), and $^{*}P < 0.05$, ER-464195-01-treated groups versus DSS-treated group at day 7 (two-tailed *t* test) (**c** and **d**).

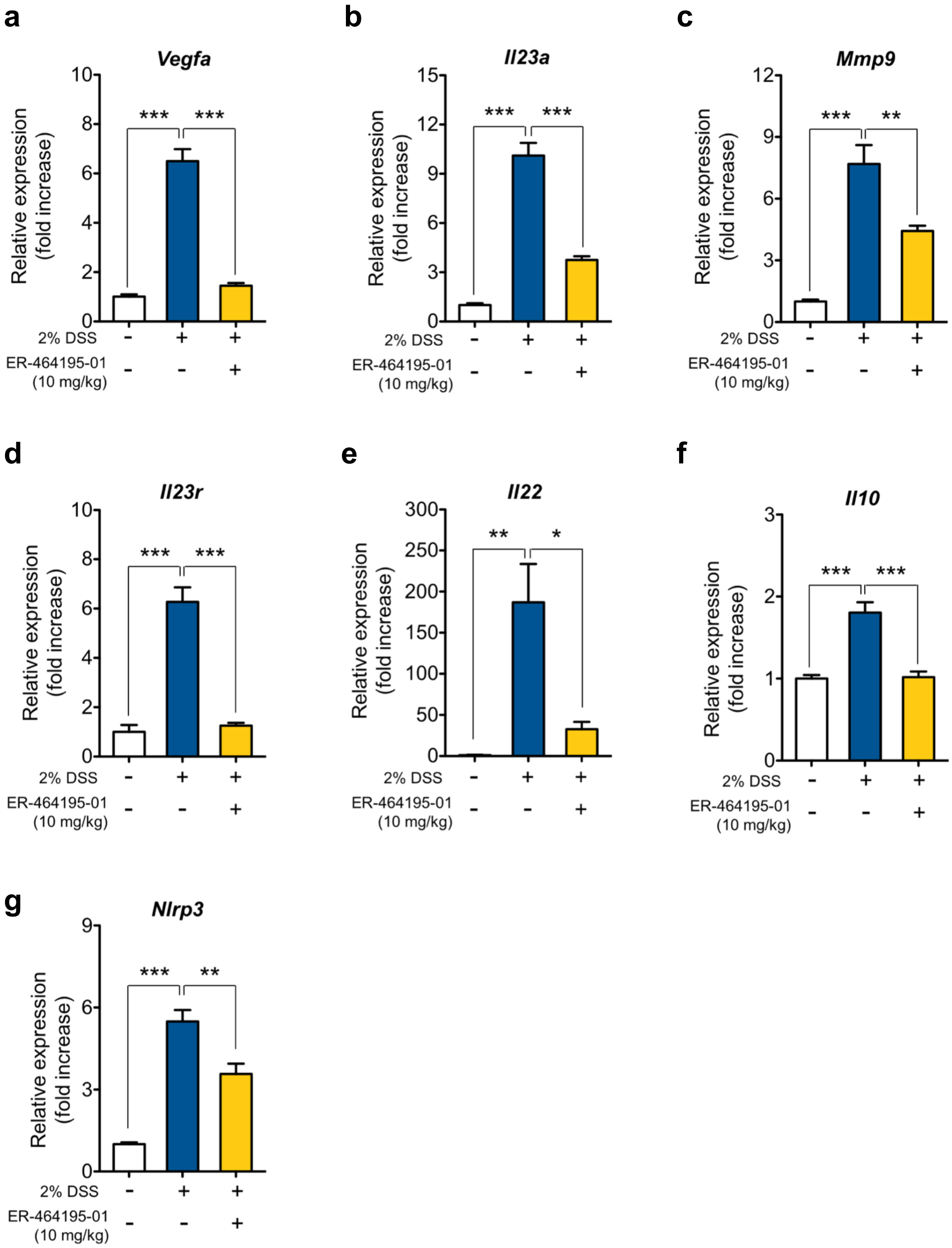

Supplementary Figure 10. Quantitative real-time PCR validation for the expression of tumor-associated genes of colons in 2% DSS-induced colitis mice. *Vegfa* (**a**), *Il23a* (**b**), *Mmp9* (**c**), *Il23r* (**d**), *Il22* (**e**), *Il10* (**f**) and *Nlrp3* (**g**). Results are given as the mean ± SEM of n = 5; $^{*}P < 0.05$, $^{**}P < 0.001$ and $^{***}P < 0.0001$ vs. DSS-treated group (two-tailed *t* test).

a

Score	Stool consistency
0	Normal
1	Loose stool^{+} (well formed pellets)
2	Loose stool^{++} (very soft but formed pellets)
3	Diarrhea^{+} (pasty stool)
4	Diarrhea^{++} (liquid stool that sticks to the anus)

$^{+}$: mild to moderate; $^{++}$: severe.
Stool consistency was evaluated blindly by assigning scores (0–4).

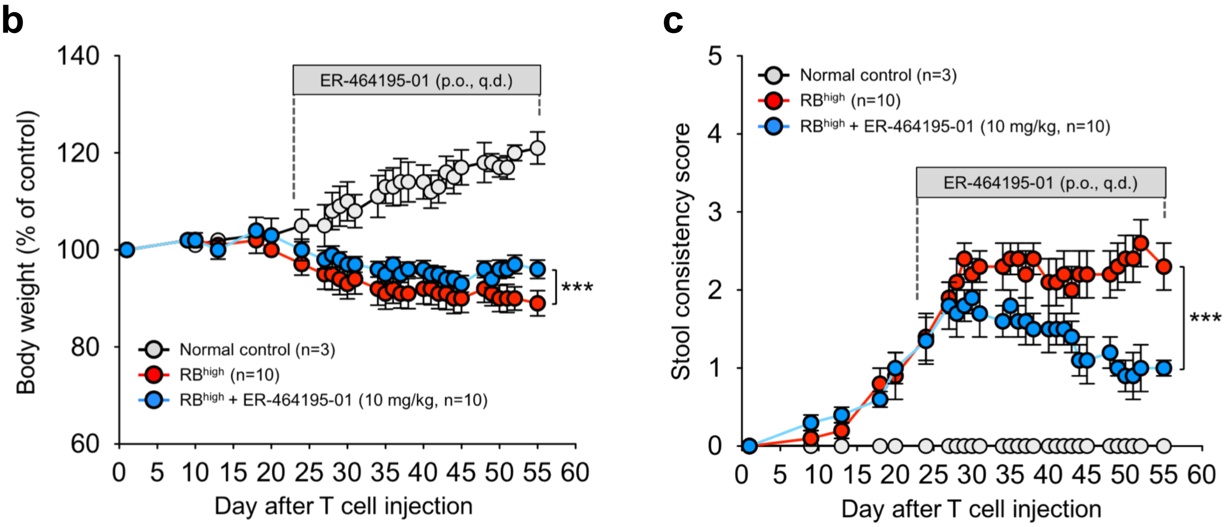

Supplementary Figure 11. Therapeutic effects of ER-464195-01 against colitis of CD4^{+}CD45RB^{high} T-cell transfer model. (**a**) Scoring method for evaluating stool consistency, (**b** and **c**) Colitis were induced by i.p. injection of CD4^{+}CD45RB^{high} T-cell into SCID mice on day 0. ER-464195-01 (p.o., q.d., days 23 to 55) improved the body weight loss (**b**) and stool consistency (**c**) at the dose of 10 mg/kg. Results are given as the mean ± SEM of n = 10; $^{***}P < 0.0001$ versus the transferred control (two-tailed *t* test).

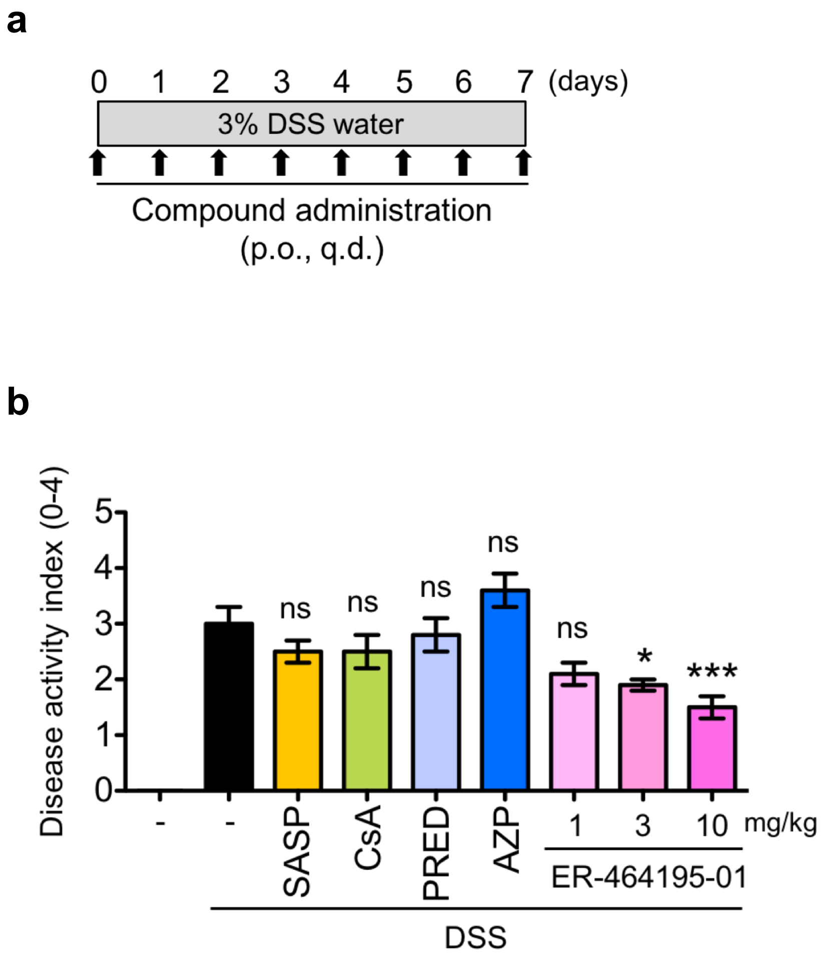

Supplementary Figure 12. Preventive effect of drugs against colitis of 3% DSS-treated mice. (**a**) Scheme of the 3% DSS mouse model. DSS-induced mice were treated with Sulfasalazine (SASP, 500 mg/kg), cyclosporin (CsA, 10 mg/kg), prednisolone (PRED, 1 mg/kg), azathioprine (AZP, 10 mg/kg) or ER-464195-01 (1, 3 and 10 mg/kg). (**b**) Disease activity index (DAI). Results are given as the mean ± SEM of n = 10; $^{*}P < 0.05$ and $^{***}P < 0.001$ vs. DSS alone group (one-way ANOVA followed by Dunnett's multiple comparison test).

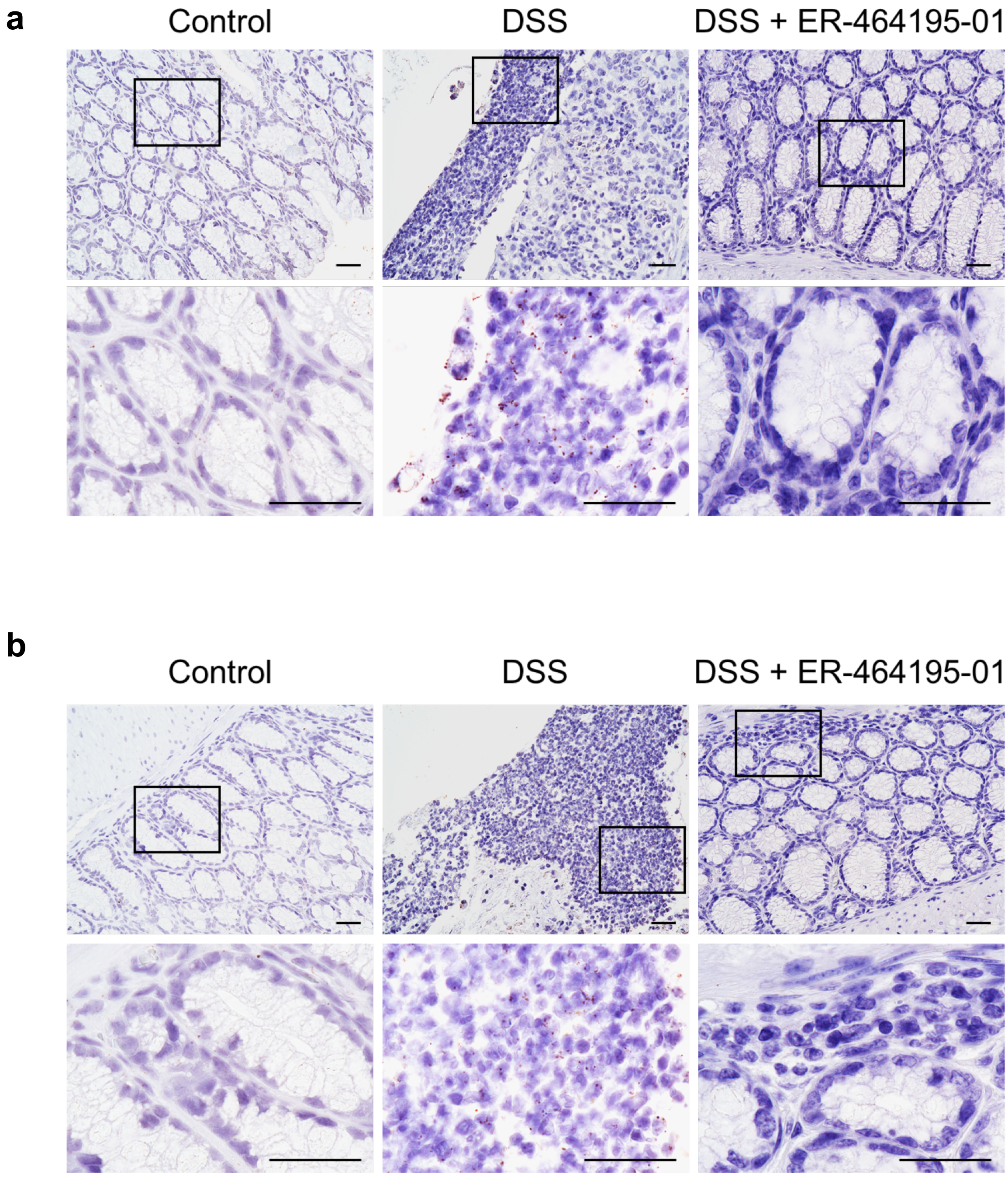

Supplementary Figure 13. *In situ* PLA assay for the CRT−ITGA4 interaction at the colonic mucosa in DSS-induced colitis mice. (**a** and **b**) Representative images of two independent experiments; control (normal water) (left), DSS treatment (middle) and the therapeutic treatment with ER-464195-01 (right). Bottom panels; enlarged boxes. Scale bars, 20 μm.

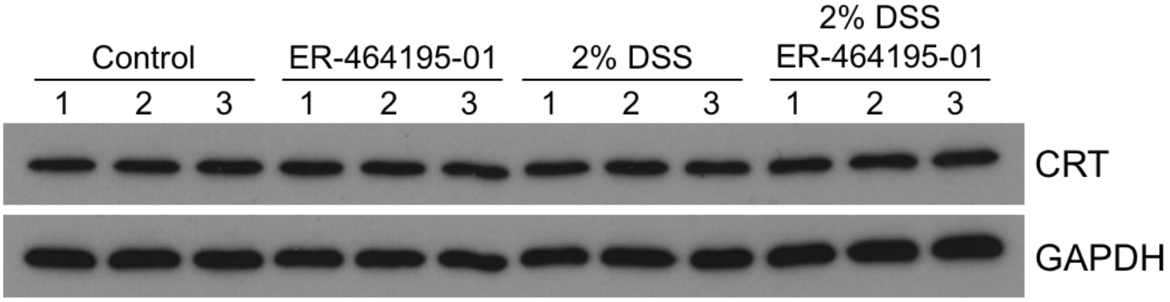

Supplementary Figure 14. Effect of ER-464195-01 on CRT expression levels of colons in 2% DSS-induced colitis mice. Representative Western blot image of CRT. GAPDH were used as loading controls.

Supplementary Table 1. Sequence alignment of α integrins (ITGAs) cytoplasmic domains (KxGFFKR).

Conserved sequence of the ITGA	Integrins	Distribution	Ligands
KVGFFKR	$\alpha_L\beta_2$	All leukocyte	ICAM-1
KAGFFKR	$\alpha_4\beta_1$	Lymphocyte, monocyte	Fibronectin, VCAM-1
	$\alpha_4\beta_7$	Lymphocyte (subpopulation)	MAdCAM-1, Fibronectin, VCAM-1
KLGFFKR	$\alpha_M\beta_2$	Neutrophil, macrophage	ICAM-1, iC3b
	$\alpha_2\beta_1$	Lymphocyte, monocyte	Collagen, Laminin
	$\alpha_5\beta_1$	Lymphocyte, monocyte	Fibronectin

Supplementary Table 2. Evaluation of DAI in DSS-induced colitis.

Score	Body weight loss (%)	Stool consistency	Bleeding
0	<0	Normal	Negative
1	≥0 - <5	Loose stool^{+}	Hemoccult positive^{+}
2	≥5 - <10	Loose stool^{++}	Hemoccult positive^{++}
3	≥10 - <15	Diarrhea^{+}	Gross bleeding^{+}
4	≥15	Diarrhea^{++}	Gross bleeding^{++}

$^{+}$: mild to moderate; $^{++}$: severe.

DAI was evaluated by a researcher blind to treatment group by assigning scores (0–4) to body weight loss, stool consistency, and bleeding. We calculated DAI using the following formula: DAI = (body weight loss score + stool consistency score + bleeding score)/3.

Supplementary Table 3. The primer sequences used in qPCR.

Gene	Forward	Reverse
Tnfa	CCAAATGGCCTCCCTCTCAT	TCCTCCACTTGGTGGTTTGC
Vegfa	CAGAAAATCACTGTGACCCTTGTT	CTTGGCTTGTCACATCTGCAA
Mmp9	TGTGCGACCACATCGAACTT	GGCACGCTGGAATGATCTAA
Il1b	CACAGCAGCACATCAACAAG	GTGCTCATGTCCTCATCCTG
Il6	CCTTCTTGGGACTGATGCTGGT	GACAGGTCTGTTGGGAGTGGTATC
Il10	GCTCTTACTGACTGGCATGAG	CGCAGCTCTAGGAGCATGTG
Il17f	CCCAGGGTCAGGAAGACA	CCGAAGGACCAGGATTTCT
Il22	GCAATCAGCTCAGCTCCTGT	CGCCTTGATCTCTCCACTCT
Il23a	CAGGGAACAAGATGCTGGAT	GGCTAGCATGCAGAGATTCC
Il23r	TGGACTTTTGTCGGGAATGG	TCGAAGTGATCTGTTAAATATCCCAA
Nlrp3	TCAGATTGCTGTGTGTGGGACTGA	AGCTCAGAACCAATGCGAGATCCT
Gapdh	TGTGTCCGTCGTGGATCTGA	TTGCTGTTGAAGTCGCAGGAG

Supplementary data 1. The identified differentially expressed genes (DEGs) among control (normal water), ER-464195-01 alone, DSS alone and DSS with ER-464195-01 group.

Excel file

SUPPLEMENTARY INFORMATION

Source data of gel images (uncropped Western blots). Red line indicates the approximate position of cropping. The indicated molecular weights refer to ladder bands.

Figure 1f

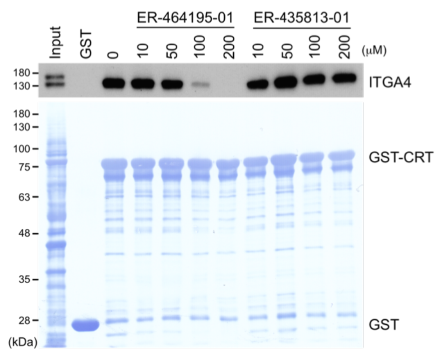

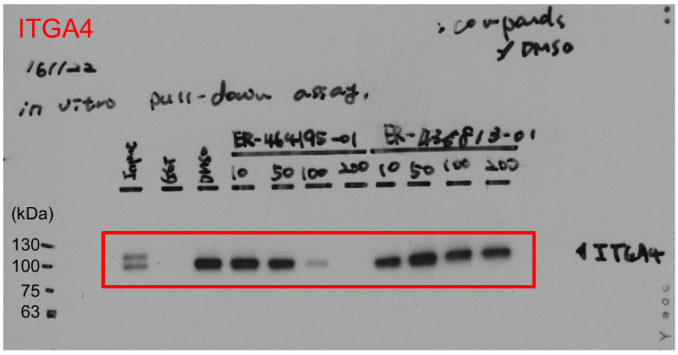

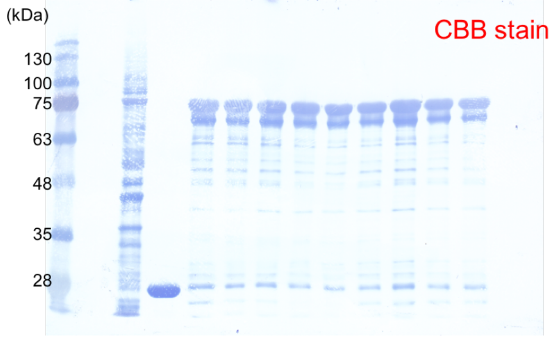

Figure 3l

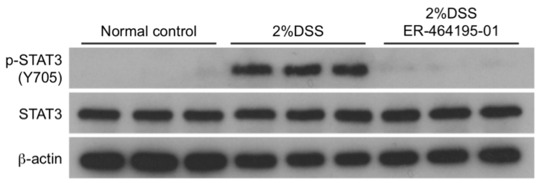

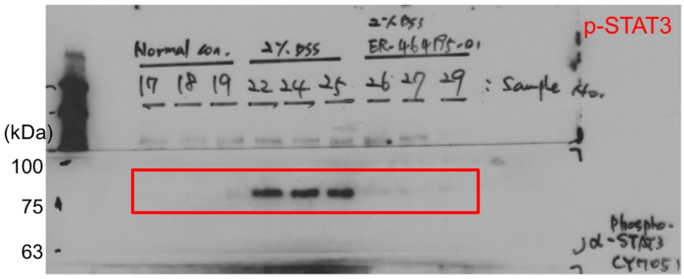

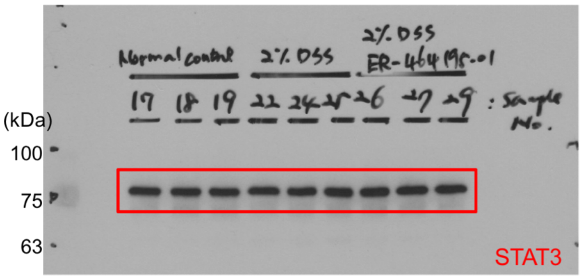

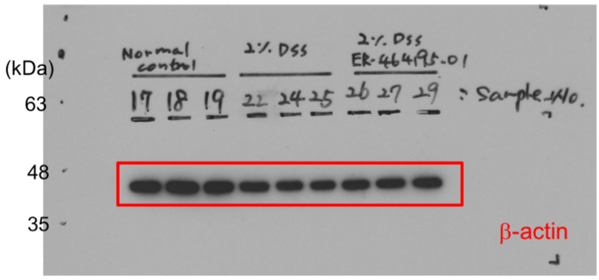

SUPPLEMENTARY INFORMATION

Source data of gel images (uncropped Western blot). Red line indicates the approximate position of cropping. The indicated molecular weights refer to ladder bands.

Supplementary Figure 14

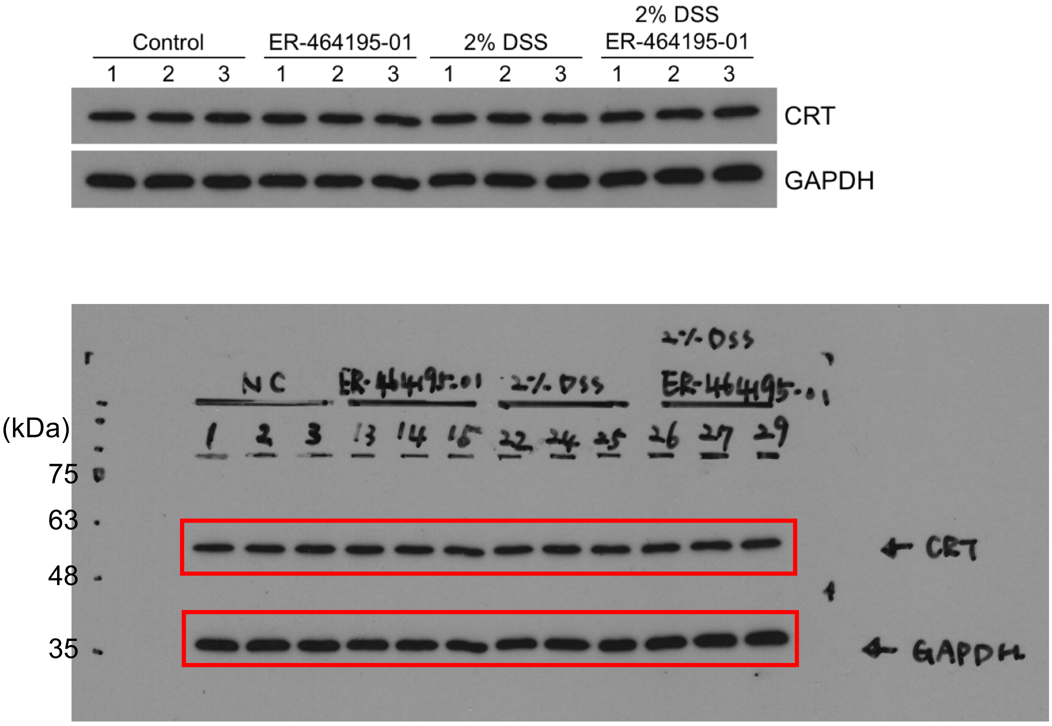

Supplement: Supplementary file 1 — Supplementary Information [file 41467_2018_4420_MOESM1_ESM.pdf]
