# Supplementary material for: Calreticulin and integrin alpha dissociation induces anti-inflammatory programming in animal models of inflammatory bowel disease
Source: Nat Commun. 2018 May 17;9:1982. doi: 10.1038/s41467-018-04420-4 (PMC5958137; doi:10.1038/s41467-018-04420-4)
Supplement: Supplementary file 3 — Description of Additional Supplementary Files [file 41467_2018_4420_MOESM3_ESM.pdf]

Description of Additional Supplementary Files

File Name: **Supplementary Data 1**
Description: The identified differentially expressed genes (DEGs) among control (normal water), ER-464195-01 alone, DSS alone and DSS with ER-464195-01 group.
